# Big Five personality traits in the workplace: Investigating personality differences between employees, supervisors, managers, and entrepreneurs

**DOI:** 10.3389/fpsyg.2023.976022

**Published:** 2023-03-28

**Authors:** Weixi Kang, Kreisha Lou Guzman, Antonio Malvaso

**Affiliations:** ^1^Imperial College London, London, United Kingdom; ^2^R&D, Macro Health Research Organization Inc., Quezon City, Philippines; ^3^Department of Brain and Behavioral Sciences, University of Pavia, Pavia, Italy

**Keywords:** personality, employee, supervisor, manager, entrepreneur, work space

## Abstract

Personality relates to employment status. Previous studies have mainly compared the difference between entrepreneurs and managers. It remains unknown how personalities differ in entrepreneurs, managers, supervisors, and employees. In this research, we answer the questions by analyzing data from Understanding Society: the UK Household Longitudinal Study (UKHLS) that consisted of 2,415 entrepreneurs, 3,822 managers, 2,446 supervisors, and 10,897 employees. By using a multivariate analysis of variance (MANOVA) and ANOVA, we found that employment status has a significant multivariate effect on personality traits (*F*(5, 17,159) = 172.51, *p* < 0.001) after taking account into demographics. Moreover, there were also significant univariate effects for Neuroticism (*F*(3,19502) = 16.61, *P* < 0.001), Openness (*F*(3,19502) = 3.53, *P* < 0.05), Agreeableness (*F*(3,19502) = 66.57, *P* < 0.001), Conscientiousness (*F*(3,19502) = 16.39, *P* < 0.001), and Extraversion (*F*(3,19502) = 31.61, *P* < 0.001) after controlling for demographics. Multiple comparisons revealed that entrepreneurs are characterized by low Neuroticism, high Openness, high Conscientiousness, and high Extraversion while managers had low Neuroticism, low Agreeableness, high Openness, high Conscientiousness, and high Extraversion. Finally, supervisors are associated with high Conscientiousness. Implications and limitations are discussed.

## Introduction

Criterion-related validity studies strongly supported the role of personality in predicting employee job performance (Ones et al., 2007; Chamorro-Premuzic and Furnham, 2010). Literature agrees that there is a significant relationship between personality and job performance across all occupational groups, managerial levels, and performance outcomes (Barrick and Mount, 1991; Hurtz and Donovan, 2000; Barrick et al., 2001). Although higher Conscientiousness and lower Neuroticism were associated with higher job performance across most types of jobs, the relationship between Extraversion, Openness, and Agreeableness with job performance was found to be more context-dependent (Barrick et al., 2001). Thus, it is important to understand how personality differs in different job positions.

Over the years, more and more people have found success in creating their businesses and working on their terms. With the number of successful entrepreneurs on the rise, researchers have become more interested in specific characteristics of entrepreneurs and how they affect their performance (Kerr et al., 2018). A notable number of studies comparing the differences in Big Five personality traits (i.e., Openness, Conscientiousness, Extraversion, Agreeableness, and Neuroticism) between entrepreneurs and managers emerged between 1960 and 2000 (Kerr et al., 2018). Managers were often compared to entrepreneurs (e.g., Zhao and Seibert, 2006), given the need of both groups to direct workers and manage multiple tasks. Both are crucial positions crucial in the company’s operations, but their roles are completely different. An entrepreneur is described as an individual who is “instrumental to the conception of the idea of an enterprise and its implementation” (Kets de Vries, 1996) and “an innovator and a catalyst of change who continuously does things that were not done before and do not fit established societal patterns” (Schumpeter, 1965). Meanwhile, a manager is defined as “the one who sets goals, plans and organizes the activities, motivates human resources, and controls the overall procedures.” (Tovmasyan, 2017). Another important player in the organizational structure is the supervisor. According to Stevens and Ash (2001), the supervisor is responsible for ensuring that the work of his subordinates is completed on time and at a satisfactory level of quality. Although the terms’ manager and supervisor are sometimes used interchangeably with managers, they are not the same. Managers are higher-level and higher-paid leaders whereas supervisors are closer to day-to-day activities of their teams to ensure the manager’s goals are met.

These observed characteristic differences in employment status are attributed to the “attraction-selection-attrition model” by Schneider (1987). According to this model, “first, individuals are attracted to jobs commensurate with their personality traits (i.e., attraction). Second, organizational selection procedures result in the selection of individuals with similar personality scale scores for a particular job (i.e., selection). Finally, individuals who take jobs to which personality traits are not suited are more likely to leave their jobs (i.e., attrition)” (Ones and Viswesvaran, 2003).

Specifically, combined evidence from the meta-analysis conducted by Zhao and Seibert (2006) reported that entrepreneurs were more open to experience, more conscientious, less agreeable, less neurotic, and but have similar levels of Extraversion compared to managers. However, many individual studies showed different patterns. One example is from a Canadian survey of 218 entrepreneurs and managers by Envick and Langford (2000), and they found that entrepreneurs were significantly less conscientious, less agreeable, and less extroverted than managers.

Entrepreneurs were also consistently found to be more open than managers. Researchers hypothesized that an entrepreneur is likely to be attracted to constantly changing environments and the novelty of new challenges in a business venture (Zhao and Seibert, 2006; Kerr et al., 2018). Individuals who thrived on challenges and novel environments presented creative solutions, business models, and products, and the Openness of entrepreneurs may help these functions (Kerr et al., 2018). Meanwhile, managers are usually chosen by their superiors to execute and deliver high-quality results for a set of directives rather than for seeking novel solutions. Thus, it is hypothesized that an entrepreneur’s environment and job requirements might be more suitable for those who were more open (Kerr et al., 2018).

Zhao and Seibert (2006) suggested that higher Conscientiousness, which is a composite of achievement motivation and dependability, is the most significant difference between entrepreneurs and managers. Their study also found that entrepreneurs and managers are similar in dependability, but entrepreneurs score significantly higher than managers in the achievement motivation facet. A meta-analysis by Collins et al. (2004) concluded that individuals who pursue entrepreneurial careers were significantly higher in achievement motivation than individuals who pursue other types of careers. Stewart and Roth (2007) similarly concluded that entrepreneurs have a higher need for achievement than managers. It is often hypothesized that achievement-oriented individuals set goals, maintain high standards, and have a strong sense of ownership. In contrast, there is insufficient evidence on whether entrepreneurs score higher than managers on Extraversion. Extraversion is a trait that measures the extent to which one is dominant, energetic, active, talkative, and enthusiastic (Costa and McCrae, 1992). Several studies found that Extraversion is more fundamental for entrepreneurs than managers since entrepreneurs act as salespeople for their ideas to investors, partners, employees, and customers. However, no reliable difference was observed in the literature according to Zhao and Seibert (2006). Further, Envick and Langford (2000) found that entrepreneurs are less extroverted than managers, suggesting that many entrepreneurs may run small businesses from their homes to be away from large bureaucracies that demand one to be relentlessly sociable.

Thus, although the personality differences between entrepreneurs and managers have been extensively studied and compared, much less is known about how personality would differ in entrepreneurs, managers, and supervisors from normal employees. Moreover, previous studies used a small sample size, which could be biased due to their reduced power. Understanding the personality differences between different employment statuses is important because understanding the personality trait differences between different employment statuses may have the potential to contribute to the established personality-job choice-job performance relationship, and thus contribute to employee selection. The aim of our study is to understand the personality difference between them by analyzing data on a large scale. We hypothesized that Openness and Conscientiousness are positively related to the employment status hierarchy (i.e., employee- > supervisor- > manager- > entrepreneur), Neuroticism and Agreeableness are negatively related to the employment status hierarchy, and Extraversion has little association with the employment status hierarchy.

## Materials and methods

### Sample and data collection

Data were from Understanding Society: the UK Household Longitudinal Study (UKHLS), which has been collecting annual information from the original sample of UK households since 1991 (when it was previously known as The British Household Panel Study (BHPS). Data were ethically collected from this sample from 2011 to 2012. This data collection has been approved by the University of Essex Ethical Committee by letter dated 17 December 2010. Samples included (1) The General Population Sample (GPS), which is a clustered and stratified probability sample of approximately 24, 000 households living in the Great Britain and a sample of approximately 2000 households in the Northern Ireland in 2009, (2) The Ethnic Minority Boost Sample (EMBS), which consists of approximately 4000 households chosen from areas with high ethnic minorities, and (3) The British Household Panel Survey sample (BHPS), which is consisted of around 8000 households. Please refer to Lynn (2009) for more details. Each household is visited each year to collect relevant information. Interviews are conducted face-to-face in participants’ homes by trained interviewers or they completed a survey online. We excluded participants who were under the age of 18 or who were above the age of 99, and those who had missing fields in relevant variables. Thus, a total number of 19,580 participants remained in our analysis from the original 49,693 participants.

### Measurement and analysis

Personality was measured using the 15-item version (3 items for each personality trait) of the Big Five Inventory with a Likert scale ranging from 1 (“disagree strongly”) to 5 (“agree strongly”). Personality scores were reversed when appropriate. The mean scores averaged across the three items for assessing each personality trait were used to represent scores for each personality trait. These shorter forms of personality measures have been approved to have good internal consistency, test-rest reliability, and convergent and discriminant validity (Hahn et al., 2012; Soto and John, 2017). Participants also responded to questions regarding if they are entrepreneurs, managers, supervisors, or employees if they were workers. Demographics information was collected from participants as well (Table 1). All analyses were conducted using a customized script on MATLAB 2018a. We used the mean scores of relevant items to represent each personality trait. A multivariate analysis of variance (MANOVA) and ANOVA were used to see the effect of employment status on personality traits in general and in detail with employment status and demographics as predictors. A multiple comparison test was used to assess the specific differences in each personality trait in different employment statuses.

**TABLE 1:** Descriptive statistics of sociodemographic variables and personality traits.

	Mean	S.D.
Age	41.38	13.04
Neuroticism	3.53	1.37
Agreeableness	5.62	1.02
Openness	4.67	1.23
Conscientiousness	5.58	1.03
Extraversion	4.66	1.27
	**N**	**%**
**Sex**		
Male	9,167	46.82
Female	10,413	53.18
**Total net personal income**		
< = 1000	5,154	26.32
> 1000 & < = 2000	9,054	46.24
>2000	5,372	27.44
**Highest educational qualification**		
Below college	12044	61.51
College	7536	38.49
**Legal marital status**		
Single	8991	45.92
Married	10589	54.08
**Employment status**		
Entrepreneur	2415	12.33
Manager	3822	19.52
Supervisor	2446	12.49
Employee	10897	55.65

## Results

Demographics can be found in Table 1. Employment status had a significant multivariate effect on personality traits (*F*(5, 17159) = 172.51, *p* < 0.001) after taking account into demographics. Moreover, there were also significant univariate effects for Neuroticism (*F*(3,19502) = 16.61, *P* < 0.001), Openness (*F*(3,19502) = 3.53, *P* < 0.05), Agreeableness (*F*(3,19502) = 66.57, *P* < 0.001), Conscientiousness (*F*(3,19502) = 16.39, *P* < 0.001), and Extraversion (*F*(3,19502) = 31.61, *P* < 0.001) after controlling for demographics (Table 2).

**TABLE 2:** The results of the ANOVA for A. Neuroticism, B. Agreeableness, C. Openness, D. Conscientiousness, and E. Extraversion respectively.

Variables	Sum Sq.	d. f.	Mean Sq.	F	Prob > F
**A. Neuroticism**					
Age	392	69	5.68	3.21	<0.001
Sex	1218.9	1	1218.9	689.13	<0.001
Personal net income	67.6	2	33.78	19.10	<0.001
Highest educational qualification	1.3	1	1.31	0.74	0.39
Marital status	5.8	1	5.82	3.29	0.07
Employment status	88.1	3	29.38	16.61	<0.001
Error	34493.9	19502	1.77		
Total	36918.6	19579			
**B. Agreeableness**					
Age	132.6	69	1.92	1.93	<0.001
Sex	473.8	1	473.81	475.24	<0.001
Personal net income	16.7	2	8.34	8.37	<0.001
Highest educational qualification	0.5	1	0.50	0.51	0.48
Marital status	0.8	1	0.78	0.78	0.38
Employment status	10.6	3	3.52	3.53	<0.05
Error	19443.5	19502	1.00		
Total	20185.4	19579			
**C. Openness**					
Age	199.5	69	2.89	2	<0.001
Sex	85	1	85.04	58.72	<0.001
Personal net income	5.3	2	2.67	1.84	0.16
Highest educational qualification	529.3	1	529.30	365.52	<0.001
Marital status	47.1	1	47.14	32.55	<0.001
Employment status	289.2	3	96.40	66.57	<0.001
Error	28240.3	19502	1.45		
Total	29653.7	19579			
**D. Conscientiousness**					
Age	491.2	69	7.12	7.14	<0.001
Sex	310.3	1	310.33	311.15	<0.001
Personal net income	12.3	2	6.14	6.15	<0.001
Highest educational qualification	19	1	19.03	19.08	<0.001
Marital status	7.5	1	7.54	7.56	<0.01
Employment status	49	3	16.35	16.39	<0.001
Error	19450.2	19502	1.00		
Total	20668.6	19579			
**E. Extraversion**					
Age	354.5	69	5.14	3.26	<0.001
Sex	536.8	1	536.83	340.56	<0.001
Personal net income	34.8	2	17.38	11.03	<0.001
Highest educational qualification	68.6	1	68.57	43.50	<0.001
Marital status	0	1	0.04	0.02	0.88
Employment status	149.5	3	49.84	31.61	<0.001
Error	30741.1	19502	1.58		
Total	31713.3	19579			

**FIGURE 1:**
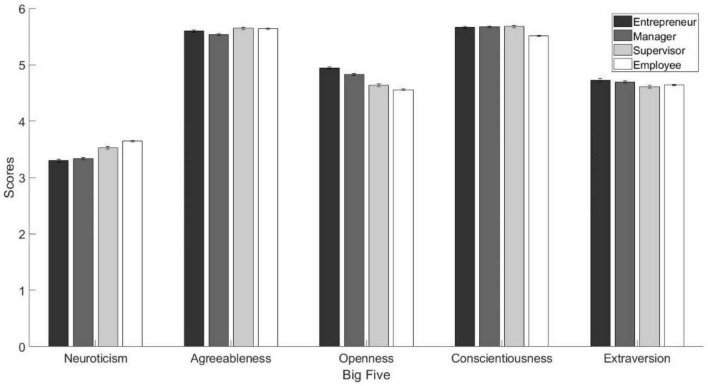
The bar graph shows differences in personality traits between different employment statuses with standard error.

Multiple comparison tests showed that entrepreneurs are less neurotic than normal employees (mean difference = −0.16, [95% CI: −0.24, −0.08], *p* < 0.001). Managers had lower Neuroticism scores than employees (mean difference = −0.16, [95% CI: −0.24, −0.08], *p* < 0.001) and supervisors (mean difference = −0.09, [95% CI: −0.18, 0.00], *p* < 0.05). Managers were less agreeable than supervisors (mean difference = −0.07, [95% CI: −0.14, 0.00], *p* < 0.05) and employees (mean difference = −0.06, [95% CI: −0.12, 0.00], *p* < 0.05). Regarding Openness, entrepreneurs were more open than managers (mean difference = 0.17, [95% CI: 0.09, 0.25], *p* < 0.001), supervisors (mean difference = 0.29, [95% CI: 0.20, 0.38], *p* < 0.001), and employees (mean difference = 0.37, [95% CI: 0.30, 0.45], *p* < 0.001). Similarly, managers had higher Openness scores than supervisors (mean difference = 0.20, [95% CI: 0.14, 0.27], *p* < 0.001) and employees (mean difference = 0.08, [95% CI: 0.01, 0.15], *p* < 0.05). Conscientiousness scores in entrepreneurs (mean difference = 0.11, [95% CI: 0.05, 0.16], *p* < 0.001), in managers (mean difference = 0.11, [95% CI: 0.06, 0.16], *p* < 0.001), in supervisors (mean difference = 0.11, [95% CI: 0.05, 0.17], *p* < 0.001) were significantly higher than that of in employees. Finally, entrepreneurs were more extroverted than supervisors (mean difference = 0.23, [95% CI: 0.14, 0.33], *p* < 0.001) and employees (mean difference = 0.25, [95% CI: 0.17, 0.32], *p* < 0.001). Managers were also more extraverted than supervisors (mean difference = 0.15, [95% CI: 0.07, 0.24], *p* < 0.001) and employees (mean difference = 0.17, [95% CI: 0.10, 0.24], *p* < 0.001; Figure 1).

## Discussion

Token together, our study compared the personality differences between employees, supervisors, managers, and entrepreneurs using multivariate and univariate ANOVA after controlling for demographics with multiple comparison tests to assess specific differences between groups. Our study is the first study that compared the Big Five personality differences between these groups according to the best of our knowledge although previous studies have compared this difference between entrepreneurs and managers. A detailed discussion is provided in the following paragraphs.

Results showed that entrepreneurs and managers exhibit lower Neuroticism compared to employees. These findings were consistent with existing studies suggesting entrepreneurs are less neurotic (Zhao and Seibert, 2006; Kerr et al., 2018). Lower levels of Neuroticism are described as having emotional stability that allows entrepreneurs to deal with stress and uncertainty, and develop a good working relationship with others (Etemad et al., 2013). Another study done by Yitshaki (2021) also highlighted the need for entrepreneurs to keep their emotions in control because their firm’s growth might depend on how they manage these. Similarly, managers have to be emotionally stable to fulfill management duties. However, we did not find a significant difference in Neuroticism between entrepreneurs and managers (Zhao and Seibert, 2006).

Similarly, we found a significant effect of employment status on Agreeableness. People with high Agreeableness were found to be more prosocial (Costa and McCrae, 1992), and it seems to be crucial for the success of entrepreneurs to gain external resources from other organizations with the help of maintained relationships (Street and Cameron, 2007). Specifically, we found that managers were less agreeableness than supervisors and employees. Indeed, although high Agreeableness may lead one to be considered trustworthy and build positive work relationships, it may prevent managers to drive hard bargains, look out for one’s own self-interest, and influence other people for one’s own advantage. All of these characteristics made it not desirable for managers because they may interfere with the manager’s ability to make difficult decisions which may affect subordinates and coworkers (Zhao and Seibert, 2006).

Similarly, we found a significant effect of employment status on Openness, which is a trait that has been often characterized by creativity, being attracted to changing environments, and prefer variety over routine (Kerr et al., 2018). Specifically, we found that managers were less open than supervisors and employees. Indeed, the goal of a manager is to control the whole procedure and ensure goals are met rather than being very creative and innovative, which requires less degree of Openness although managers’ Openness may be positively associated with organizational success (Kay and Christophel, 1995).

This study also found that Openness in entrepreneurs is higher than that of managers, supervisors, and employees. Specifically, entrepreneurs were more open than managers, supervisors, and employees. Similarly, managers were more open than supervisors and employees. Entrepreneurs are characterized by their emphasis on innovation (Zhao and Seibert, 2006). Creating a new venture may require the entrepreneur to come up with new or novel ideas, use creativities to solve problems that have not been encountered before, and make innovative products, business models, or strategies. Interestingly, we also found that managers are more open than supervisors and employees, which may indicate that even though enforcing the rules is important, being innovative in establishing policies and making strategies is also critical for the success of the manager as well.

Conscientiousness is described as a person’s ability to control their impulses, develop long-term goals, and consistently work on these goals to achieve them. In this study, we found that entrepreneurs, managers, and supervisors have higher Conscientiousness scores than normal employees. Despite mixed results of previous studies (Envick and Langford, 2000; Collins et al., 2004; Stewart and Roth, 2007; Cantner et al., 2011), the role of Conscientiousness is generally considered important in entrepreneurship which was stressed by Ciavarella et al. (2004) as the positive link between long-term venture survival. Additionally, Hough and Oswald (2000) reported that Conscientiousness is the strongest predictor of managerial performance. Ülgen et al. (2016) discussed the relationship between Conscientiousness and management styles and found significant effects of Conscientiousness on management styles that require rational decision-making like authoritarian, protective, supporter, and laissez-faire styles but not on the unionized styles.

We also found that Extraversion scores in entrepreneurs and managers are significantly higher than that of supervisors or employees. Individuals with Extraversion tend to be dominant, energetic, talkative, and enthusiastic (Costa and McCrae, 1992). Entrepreneurs are most likely to get involved in activities that require a high level of social skills, it is expected that they exhibit higher levels of Extraversion, which is heavily supported by our results. Thus, having jobs not requiring much interaction with other people could explain why average employees had the lower level of Extraversion among the other statuses of employment. The finding that entrepreneurs do not have higher Extraversion scores than managers seemed to be consistent with one previous study (Awwad and Al-Aseer, 2021) but contradictory to others (e.g., Zhao and Seibert, 2006).

There are some limitations in this study. First, we used cross-sectional data and all the relationships in the current study were associative, which makes it hard to identify the causal effect. Thus, it remains unclear regarding if certain personality traits cause people to be in certain employment status or if employment status causes changes in personality traits. Second, we measured employment status in general, it is unclear how personality in a different occupation and in different employment statuses would differ. For instance, a salesman’s personality could totally differ from an assembly line worker as the main activity of a salesman is to engage with other people, which requires more social skill and thus have different personality traits. Moreover, compared to personality traits, characteristics such as general or emotional intelligence, temperament or motivation, or interests and aspirations may be more important in differentiating occupational positions (McManus et al., 2003; Cheng and Furnham, 2012; Stoll et al., 2017).

This study provided novel insights and further understanding of how the Big Five personality traits vary across different employment statuses. A deeper comprehension of the connection between personality and employment status has the possibility to be useful in several practical fields. Although theories of vocational choice have found considerable application in the context of career counseling, different employment status as a career path has received less consideration in this literature. Our findings offer proof of the personality traits that set someone who is likely to be drawn to, chosen for, and stay in a different employment status. With this knowledge, people will be better able to match their strengths to the risks and opportunities presented by a professional career. The decisions made by venture capitalists, government funding organizations, and others on their support for certain employment status may be influenced, at least in part, by their own theories and models of employment status and personality. Decision-makers may become more realistic and modest in the implementation of their own implicit ideas if they are aware of the true relationship between personality and employment status. Large firms frequently work to foster innovation by choosing staff members who will act as internal entrepreneurs (intrapreneurs) and elevating them to important positions. The study’s findings can be used to create suitable selection and placement standards for such choices. Furthermore, this study has consequences for how people interested in entrepreneurship should be trained. Even though the Big Five fundamental personality traits are generally stable, many of the behaviors connected to them can be learned with experience and effort. For instance, research by Barrick et al. (1993) revealed that people who scored highly on Conscientiousness were more likely to develop and stick to goals, which was then linked to their better job performance. Both the person seeking to pursue different positions and society at large may find training intended to promote the behaviors associated with employment status to be very useful. We don’t believe that personality theory offers a comprehensive theory of employment status or even covers all the possible themes. Instead, our findings demonstrate that personality must be taken into account as one significant element in a multidimensional model of the variables, processes, and contextual factors influencing employment status and the establishment of new ventures.

## Data availability statement

Publicly available datasets were analyzed in this study. This data can be found here: https://www.understandingsociety.ac.uk.

## Ethics statement

The studies involving human participants were reviewed and approved by University of Essex. The patients/participants provided their written informed consent to participate in this study.

## Author contributions

WK: conceptualization, data curation, formal analysis, investigation, methodology, resources, software, writing – original draft, and writing – review and editing. KG: writing – original draft. AM: writing – review and editing. All authors contributed to the article and approved the submitted version.
